# Constructing 1 + 1 > 2 Photosensitizers Based on NIR Cyanine–Iridium(III) Complexes for Enhanced Photodynamic Cancer Therapy

**DOI:** 10.3390/molecules30122662

**Published:** 2025-06-19

**Authors:** Ziwei Wang, Weijin Wang, Qi Wu, Dongxia Zhu

**Affiliations:** Key Laboratory of Nanobiosensing and Nanobioanalysis at Universities of Jilin Province, Department of Chemistry, Northeast Normal University, 5268 Renmin Street, Changchun 130024, China; wangzw026@nenu.edu.cn (Z.W.); wangwj270@nenu.edu.cn (W.W.); wuq946@nenu.edu.cn (Q.W.)

**Keywords:** near infrared, Ir(III) complex, cyanine dye, photodynamic therapy

## Abstract

Photosensitizers with high singlet oxygen (^1^O_2_) generation capacity under near-infrared (NIR) irradiation are essential and challenging for photodynamic therapy (PDT). A simple yet effective molecular design strategy is realized to construct 1 + 1 > 2 photosensitizers with synergistic effects by covalently integrating iridium complexes with cyanine via ether linkages, as well as introducing aldehyde groups to suppress non-radiative decay, named **CHO−Ir−Cy**. It is demonstrated that **CHO−Ir−Cy** successfully maintains the NIR absorption and emission originated from cyanine units and high ^1^O_2_ generation efficiency from the iridium complex part, which gives full play to their respective advantages while compensating for shortcomings. Density functional theory (DFT) calculations reveal that **CHO−Ir−Cy** exhibits a stronger spin–orbit coupling constant (ξ (S1, T1) = 9.176 cm^−1^) and a reduced energy gap (ΔE = −1.97 eV) between triplet excited states (T_1_) and first singlet excited states (S_1_) compared to parent Ir−Cy or Cy alone, directly correlating with its enhanced ^1^O_2_ production. Remarkably, **CHO−Ir−Cy** demonstrates superior cellular internalization in 4T1 murine breast cancer cells, generating substantially elevated ^1^O_2_ yields compared to individual Ir−Cy/Cy under 808 nm laser irradiation. Such enhanced reactive oxygen species production translates into effective cancer cell ablation while maintaining favorable biocompatibility, significant phototoxicity and negligible dark toxicity. This molecular engineering strategy overcomes the inherent NIR absorption limitation of traditional iridium complexes and ensures their own high ^1^O_2_ generation ability through dye–metal synergy, establishing a paradigm for designing metal–organic photosensitizers with tailored photophysical properties for precision oncology.

## 1. Introduction

In recent years, more and more people have died of cancer, which has become one of the most threatening diseases in the world [1,2,3]. Clinically, feasible cancer treatment strategies usually include radiotherapy (RT), chemotherapy (CT), immunotherapy, photodynamic therapy (PDT), photothermal therapy (PTT) and the combination of various treatment techniques [4,5,6,7,8,9]. Although these technologies are successful, the complexity of most of the designs has limited their adoption on a wide scale. Therefore, there is a need to explore a simpler and more efficient approach to cancer treatment.

Compared with traditional cancer treatment methods, PDT has been favored by more and more researchers because of its minimal invasion, high specificity and negligible side effects on surrounding normal tissues [10,11]. At present, the basic research on phototherapy for tumor treatment remains highly active, while clinical translation progresses slowly, one of the main reasons being the absence of an ideal photosensitizer (PS). PDT usually requires three essential components: light source, PSs and oxygen [12,13,14,15]. Upon appropriate light irradiation, PSs will generate reactive oxygen species (ROS), which will oxidize biomolecules in cells, resulting in cytotoxicity [16]. In particular, PSs with excellent performance exert a multiplier effect in PDT treatment. However, traditional organic molecule PSs have many defects, such as poor photostability, limited intersystem crossing (ISC) efficiency and visible light energy excitation, which weaken their ability to generate ROS and thus affect the therapeutic effect [17,18].

Compared to conventional organic small molecules, iridium(III) complexes exhibit significant advantages, such as large Stokes shifts, strong spin–orbit coupling, high luminescence efficiency, long phosphorescence lifetimes and superior triplet exciton generation efficiency, making them highly promising candidates for developing high-performance photosensitizers [19,20,21]. However, traditional iridium complexes typically exhibit absorption in the ultraviolet–visible (UV–Vis) spectral range [22,23,24,25,26,27,28,29]. Due to activation via the short wavelength of external light, which is easily scattered by biological tissues, their clinical applications in PDT are severely limited [30]. In contrast, the absorption band of anthocyanin dyes can extend into the near-infrared region. Near-infrared (NIR) irradiation can achieve deeper tissue penetration, which means that, while allowing effective light to penetrate living tissues, it minimizes photodamage to healthy tissues and effectively treats deep-seated diseases [31,32,33,34,35,36]. Nevertheless, the development of NIR-activatable photosensitizers remains challenging due to the energy gap law: when the absorption band redshifts to the NIR region (corresponding to a reduced energy gap Δ*E*), the increased wavefunction overlaps between the zero-vibrational level of the excited state and the isoenergetic high-vibrational levels of the ground state, accelerating intramolecular vibrational relaxation [37,38]. This leads to enhanced non-radiative decay rates, significantly shortening the triplet excited state lifetime [39]. Consequently, the reduced energy/electron transfer time to the surrounding substrates or molecular oxygen diminishes ROS generation efficiency [40]. To counteract these limitations, vibrational decoupling strategies, such as employing rigid ligand frameworks (e.g., introducing sterically hindered aromatic rings) [41] or leveraging heavy-atom effects (e.g., incorporating bromine or iridium) [42], can suppress vibrational relaxation rates. These approaches effectively mitigate the accelerated non-radiative decay caused by reduced ΔE. However, the complexity of the often-used synthetic routes for such NIR complexes has limited their development [43,44]. Metal ions and cyanine dyes with NIR absorption effectively enhance their PTT performance through simple coordination binding but have little effect on their PDT performance [45,46]. Therefore, the rational design of iridium-based photosensitizers through simple strategies to synergistically integrate NIR absorption capability with prolonged triplet state lifetimes remains an urgent task for advancing clinical tumor PDT applications.

**Scheme 1:**
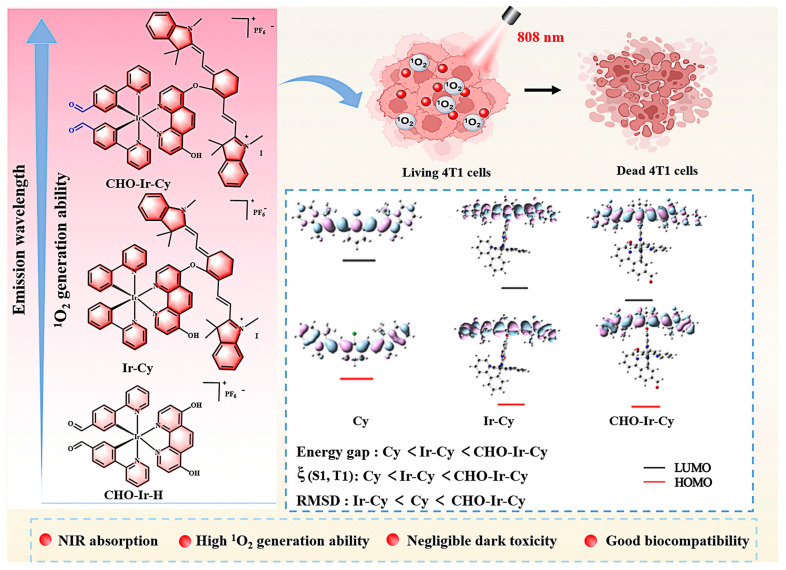
Schematic diagrams of **CHO−Ir−Cy**, Ir−Cy, Cy and PDT effects under NIR light irradiation.

**CHO−Ir−Cy**, through a simple yet effective molecular design strategy, was achieved herein by covalently conjugating iridium complexes to cyanine via ether bonds while introducing aldehyde groups to effectively suppress non-radiative decay. **CHO−Ir−Cy** retains the NIR absorption/emission of cyanine units and the high ^1^O_2_ generation efficiency of the iridium complex, thereby maximizing their complementary advantages, respectively. In this work, we developed a 1 + 1 > 2 iridium(III) complex, not only taking full advantage of both organic small molecule and transition metal complex photosensitizers but also overcoming the disadvantage of instability of cyanine, achieving better therapeutic effects than iridium complexes and cyanine separately. Aldehyde groups are introduced into cyclometal ligands to increase their ^1^O_2_ yield, which successfully overcomes the intrinsic short-wavelength excitation light defects of conventional transition metal iridium complexes. **CHO−Ir−Cy** can be efficiently taken up by 4T1 cells, and a large amount of ^1^O_2_ can be produced to kill cancer cells under the 808 nm laser irradiation (0.5 W cm^−2^). Otherwise, it also shows good biocompatibility, phototoxicity and low dark toxicity, suggesting that NIR cyanine–iridium(III) complexes have potential in photodynamic clinical treatment in the future (Scheme 1).

## 2. Results and Discussion

### 2.1. Synthesis of Ligands and Complexes

The dyes were synthesized via conventional methods [47]. ppy-Ir-H and CHO-Ir-H complexes were reacted with cyanine under dark conditions in a nitrogen (N_2_) atmosphere to yield Ir−Cy and **CHO−Ir−Cy**. The characterization data, including nuclear magnetic resonance (NMR) spectroscopy and mass spectrometry (MS), are shown in Figures S1–S6 in the Supporting Information.

### 2.2. Analysis of Photophysical Properties of Complexes

**Figure 1:**
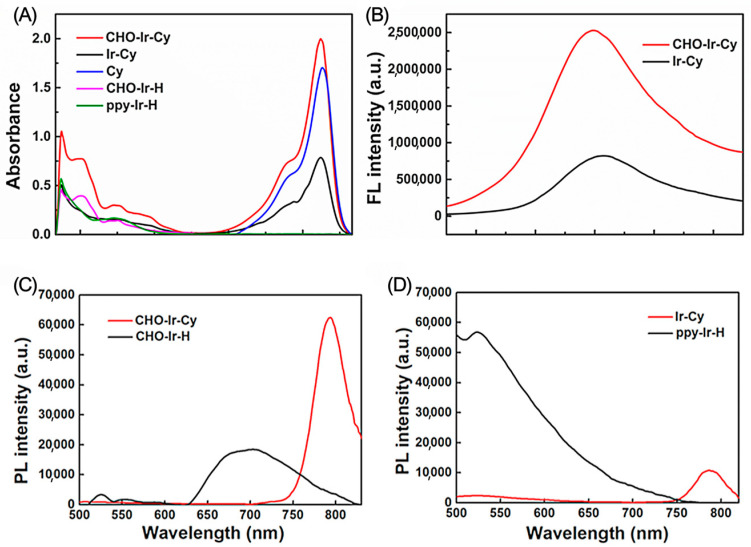
(**A**) UV–Vis absorption spectra of **CHO−Ir−Cy**, Ir−Cy, Cy, CHO-Ir-H and ppy-Ir-H (10^−5^ M) in DMSO; (**B**) The PL spectra of **CHO−Ir−Cy** and Ir−Cy (10^−5^ M) in DMSO, *λ*_ex_ = 730 nm; (**C**) The PL spectra of **CHO−Ir−Cy**, CHO-Ir-H; (**D**) Ir−Cy, ppy-Ir-H, *λ*_ex_ = 380 nm.

We tested the UV–Vis absorption spectra of **CHO−Ir−Cy**, Ir−Cy, Cy, CHO-Ir-H and ppy-Ir-H in DMSO. As shown in Figure 1A, **CHO−Ir−Cy** and Ir-Cy exhibited strong NIR absorption peaks in the 600–800 nm range. This observation indicates that these compounds retain the NIR absorption advantages of Cy while simultaneously addressing the limitation of most iridium metal complexes, which lack absorption in the NIR region. This improvement enables the use of long-wavelength NIR light as an excitation source. Furthermore, the absorption spectra of **CHO−Ir−Cy** and Ir−Cy also preserve the characteristic absorption features of metal iridium complexes, suggesting that they combine the strong NIR absorption of small organic photosensitizers with the ISC capability of transition metal complexes—a critical property for effective PDT. The molar absorption coefficients of **CHO−Ir−Cy** and Ir−Cy at 808 nm are 100,019 M^−1^ cm^−1^ and 38,584 M^−1^ cm^−1^, respectively. These high values demonstrate excellent light-harvesting efficiency in the NIR region, which is essential for achieving robust ^1^O_2_ generation. Additionally, the emission maxima of **CHO−Ir−Cy** and Ir−Cy are centered near 800 nm (Figure 1B). Notably, the incorporation of Cy successfully redshifted the emission of the iridium complexes to wavelengths > 750 nm (Figure 1C,D), thereby aligning their photophysical properties with the requirements of deep-tissue PDT. These photophysical data conclusively demonstrate that the NIR absorption of iridium complexes is effectively enhanced through the molecular design of **CHO−Ir−Cy** and Ir−Cy. This advancement overcomes the limited tissue penetration depth of visible light by utilizing NIR excitation, thereby improving their suitability for PDT applications.

### 2.3. Density Functional Theory Calculations

**Figure 2:**
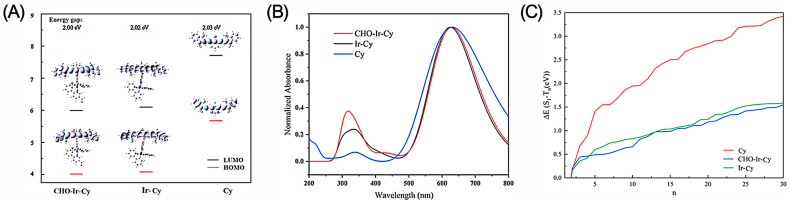
(**A**) HOMO–LUMO energy level distribution and energy gap of **CHO−Ir−Cy**, Ir-Cy and Cy; (**B**) Theoretical absorption spectra of **CHO−Ir−Cy**, Ir−Cy and Cy; (**C**) Energy difference trend diagram for **CHO−Ir−Cy**, Ir−Cy and Cy.

The computational analysis reveals that the HOMO and LUMO of Cy, Ir−Cy and **CHO−Ir−Cy** are predominantly localized on the cyanine moiety (Figure 2A). Notably, the HOMO and LUMO energy levels of molecules containing Ir(III) complexes are significantly lowered, accompanied by a narrower bandgap compared to the pure dye: Cy (2.03 eV) > Ir−Cy (2.02 eV) > **CHO−Ir−Cy** (2.00 eV). This observation aligns with the conclusion derived from optical spectra (Figure 2B), confirming that Ir−Cy and **CHO−Ir−Cy** retain the intrinsic optical characteristics of the parent cyanine. These optimized electronic properties enhance light-harvesting efficiency under long-wavelength irradiation and increase the intramolecular charge transfer efficiency, demonstrating their potential as phototherapeutic agents for deep-tissue applications.

A systematic analysis of the excited-state properties of Ir−Cy and **CHO−Ir−Cy** was performed. At the TD-B3LYP/6-31G(d) SMD (solvent = DMSO) level, the calculated excitation energies, oscillator strengths and dominant transition components are summarized in Tables S2 and S3. The strong absorption in the long-wavelength region for the S_1_ of **CHO−Ir−Cy** and Ir−Cy closely resembles that of Cy, primarily attributed to the contribution of the cyanine fragment. Analysis of the energy gap diagrams between S_1_ and triplet excited states (T_1_) reveals that the optical properties of all three molecular materials at lower energy levels originate predominantly from the dye moiety, preserving the luminescent characteristics of the parent dye molecule. The Ir(III) complex unit significantly influences higher-energy excited-state properties; however, transitions involving high-energy excitons require substantial energy consumption due to elevated energy barriers. The experimental data in Figure 1C,D demonstrate that the luminescence of the Ir complex moiety in **CHO−Ir−Cy** and Ir−Cy is largely quenched, while the dye molecule’s emission remains unaltered, thereby corroborating the theoretical predictions.

**Figure 3:**
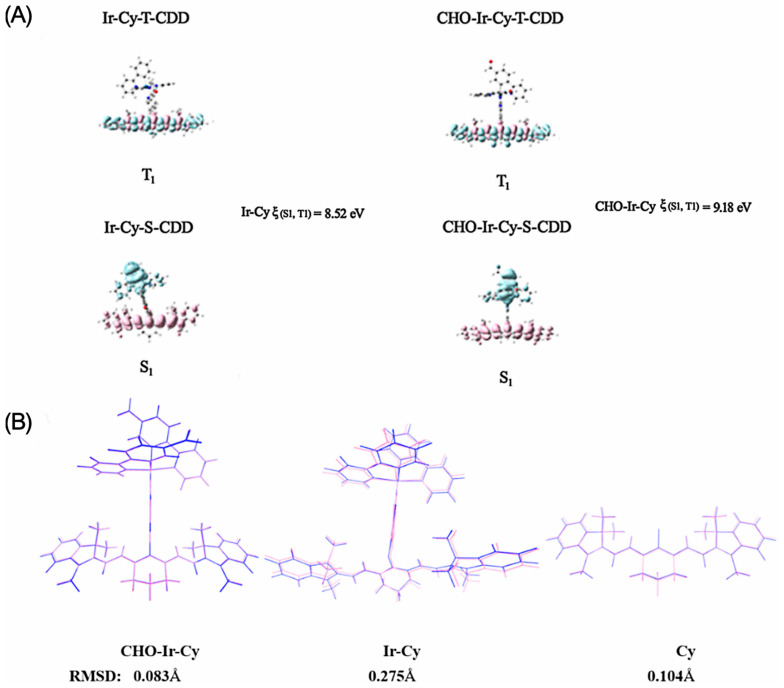
(**A**) The transition charge density differences (CDDs) for singlet (S_n_) and triplet (T_n_) excited states in **CHO−Ir−Cy** and Ir−Cy; (**B**) Schematic diagram of the configuration changes and RMSD values of S_0_ and T_1_ structures of **CHO−Ir−Cy**, Ir−Cy and Cy after relaxation.

As shown in Figure 2C, compared to Cy, Ir-Cy and **CHO−Ir−Cy** exhibit more densely populated excited-state energy levels, generating a greater number of excited states within the 4.00 eV range. This facilitates enhanced exciton generation efficiency in Ir−Cy and **CHO−Ir−Cy** and improves the ISC capability between their singlet and triplet states. Analysis of the spin–orbit coupling (SOC) constants (Table S4) and charge density difference (CDD) diagrams reveals (Figure 3A) that the introduction of the aldehyde group does not alter the intrinsic luminescent nature of the Ir−Cy material. The ξ (S1, T1) of **CHO−Ir−Cy** is 459 times greater than that of **Cy** and also 1.08 times greater than that of Ir-Cy due to the low heavy-atom participation in both states. The larger SOC value in **CHO−Ir−Cy**, combined with smaller energy gaps at higher energy levels, facilitates the ISC from S_1_ to T_1_, which is critical for enhancing the performance of **CHO−Ir−Cy** as a photosensitizer.

The localized charge-transfer transition (^3^LC) within the cyanine component ultimately leads to the formation of the lowest S1. In this localized excited state, there is no participation from the Ir complex moiety, which explains the luminescence quenching of the Ir complex unit in **CHO−Ir−Cy** and Ir−Cy. This also indicates that, although the Ir complex moiety generates longer-lived triplet excitons, **CHO−Ir−Cy** and Ir−Cy still exhibit long-wavelength absorption and NIR emission originating from the Cy fragment. Consequently, the efficient ISC capability between the singlet (S_n_) and triplet (T_n_) excited states of the Ir complex moiety enables these materials to populate more triplet excitons, thereby significantly enhancing the ^1^O_2_ generation efficiency of Ir−Cy and **CHO−Ir−Cy**.

To evaluate the impact of aldehyde group introduction on molecular energy loss, we calculated the root mean square deviation (RMSD) values of structural relaxation for the S_0_ (ground state) and T_1_ geometries in **CHO−Ir−Cy** and Ir−Cy: 0.083 Å and 0.275 Å, respectively (Figure 3B). The lower RMSD value for **CHO−Ir−Cy** indicates reduced non-radiative energy loss due to the aldehyde group, which favors improved photosensitizer performance. These DFT calculation results align with experimental observations, further demonstrating that rational molecular design strategies can integrate traditional organic photosensitizers with Ir(III) complexes to develop NIR-light-activated Ir(III)-based photosensitizers.

#### 2.3.1. Analysis of Photostability Examination in Solution

**Figure 4:**
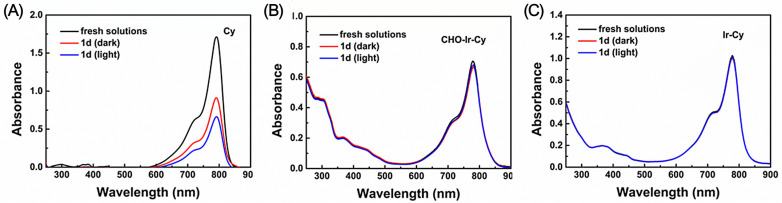
UV–Vis absorption spectra of (**A**) Cy, (**B**) **CHO−Ir−Cy** and (**C**) Ir−Cy under different treatment conditions: (i) freshly prepared solution (baseline), (ii) dark-stored for 24 h and (iii) light-exposed for 24 h.

Excellent optical properties are a critical feature of high-performance photosensitizers. Studies have shown that cyanine in aqueous solutions tends to aggregate, leading to fluorescence quenching, and it is also prone to photodegradation under light exposure [48]. Cy, as a near-infrared cyanine derivative, suffers from similar limitations. To investigate the photostability of the complexes **CHO−Ir−Cy** and Ir−Cy, we treated both the complexes and free Cy under three conditions and monitored their UV spectral changes: (1) freshly prepared solution (baseline); (2) dark-stored for 24 h; (3) light-exposed for 24 h. As shown in Figure 4A, the absorption intensity of Cy decreased significantly under both dark and light conditions, with a more pronounced decline under light exposure, indicating its inherent photosensitivity and susceptibility to degradation. In contrast, the absorption intensities of **CHO−Ir−Cy** and Ir−Cy solutions remained nearly unchanged under the same treatments (Figure 4B,C). These UV absorption spectral data demonstrate that the covalent integration of Cy with the Ir(III) complex effectively enhances the photostability of the cyanine. This improvement plays a critical role in ensuring the superior performance of the resulting Ir(III)-based photosensitizers with NIR light absorption capabilities.

#### 2.3.2. Analysis of Singlet Oxygen Production Capacity in Solution

The production of ^1^O_2_ is very important in photodynamic cancer therapy. In this text, 1,3-diphenylisobenzofuran (DPBF) was employed as an ^1^O_2_ indicator, and its absorbance change at 415 nm was detected via ultraviolet–visible absorption spectrum to explore the ^1^O_2_ generation ability of the complex in solution. We measured the ^1^O_2_ production capacity of **CHO−Ir−Cy** and Ir−Cy in solution. The following two groups were used as blank control groups: (1) **CHO−Ir−Cy**/Ir-Cy + DPBF + no illumination; (2) DPBF + illumination.

**Figure 5:**
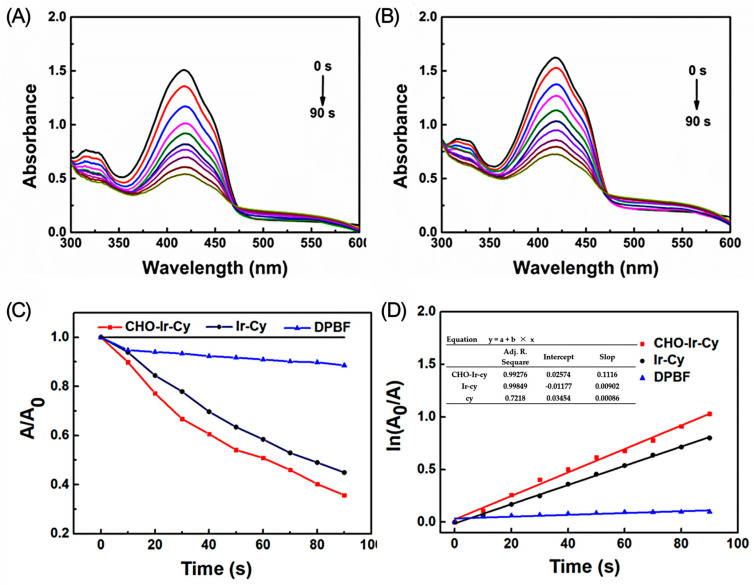
(**A**) UV absorption spectra of DPBF under 730 nm light irradiation in the presence of **CHO−Ir−Cy** and (**B**) Ir−Cy at different times; (**C**) The degradation rate of DPBF at 415 nm in the presence of different PSs at different times under 730 nm illumination; (**D**) Time-dependent ^1^O_2_ generation kinetics curve. A_0_ = maximum absorbance before illumination; A = maximum absorbance after illumination.

As shown in Figures S7 and S8, the absorption intensity of the complex + DPBF + no illumination group and the DPBF + illumination group did not decrease obviously at 415 nm, indicating that **CHO−Ir−Cy** and Ir−Cy did not generate ^1^O_2_ under no-illumination conditions. However, as shown in Figure 5A,B, after 90 s of illumination, the absorption intensity of the **CHO−Ir−Cy** and Ir−Cy complex + DPBF + illumination group decreased obviously at 415 nm, indicating the generation of effective ^1^O_2_. The ^1^O_2_ generation rates of the complexes **CHO−Ir−Cy** and Ir−Cy conform to the first-order kinetic equation (Figure 5C,D), and the order of slope changes is **CHO−Ir−Cy** (0.01116) > Ir−Cy (0.00912). The greater the slope, the faster the degradation rate of DPBF and the stronger its ^1^O_2_ generation capacity. It can be seen that **CHO−Ir−Cy** shows more excellent ^1^O_2_ production ability, which is also consistent with the results calculated using the density functional theory. In addition, according to the literature report [49], we calculated that the ^1^O_2_ yields of **CHO−Ir−Cy** and Ir−Cy were 67% and 52%, respectively, based on methylene blue (the ^1^O_2_ yield was 52%). The excellent ^1^O_2_ yield enabled **CHO−Ir−Cy** to be used as an efficient PS in PDT application research.

### 2.4. Study of Endocytosis Behavior

**Figure 6:**
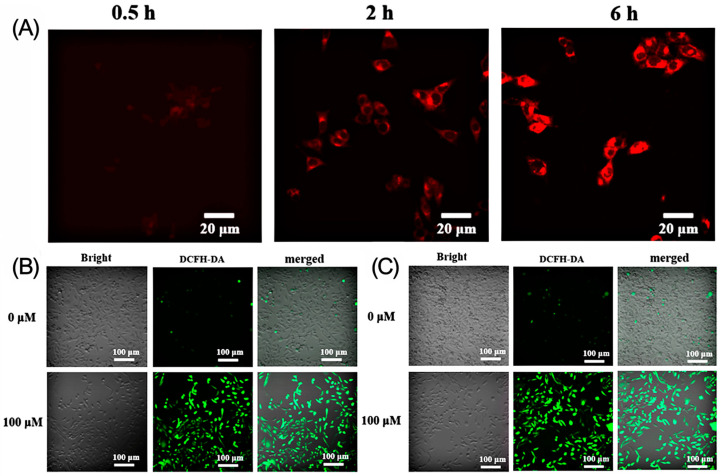
(**A**) CLSM images of 4T1 cells treated with **CHO−Ir−Cy** for 0.5 h, 2 h and 6 h. Fluorescence images of ^1^O_2_ produced by (**B**) **CHO−Ir−Cy** and (**C**) Ir−Cy in cells under different conditions with DCFH-DA as an indicator.

We investigated the ability of 4T1 cells to take up **CHO−Ir−Cy**. 4T1 cells were incubated with **CHO−Ir−Cy** for 0.5 h, 2 h and 6 h, respectively. With the incubation time prolonged, the red fluorescence signal generated in the cells gradually increased, indicating that the uptake process of the cells was time-dependent. As shown in Figure 6A, the red fluorescence emitted by **CHO−Ir−Cy** can be seen in the cells, indicating that **CHO−Ir−Cy** can be effectively endocytosed by 4T1 cells, and it also shows that **CHO−Ir−Cy** has good biocompatibility. Effective cell uptake and good biocompatibility provide a solid foundation for **CHO−Ir−Cy** as a photosensitizer to be better used in photodynamic cancer therapy.

### 2.5. Analysis of Intracellular Singlet Oxygen Production Capacity

UV–Vis absorption spectra confirmed that **CHO−Ir−Cy** and Ir−Cy had good ^1^O_2_ production ability in solution, and we further verified their ^1^O_2_ production ability in cells. The production of ^1^O_2_ in cells was detected using 2′,7′-dichlorodihydrofluorescein diacetate (DCFH-DA). Cells without photosensitizers were also used as a blank control group. As shown in Figure 6B,C, after being irradiated with an 808 nm laser for 2 min, the blank control group basically did not observe the appearance of green fluorescence, which ruled out the interference of other substances in the cell on the DCFH-DA detection indicator. When the photosensitizer content is 100 μM, the appearance of green fluorescence indicates that a large amount of ^1^O_2_ is produced in the cell. These results show that **CHO−Ir−Cy** and Ir−Cy can be used as effective PSs in PDT research.

### 2.6. Cytotoxicity Research and Analysis

**Figure 7:**
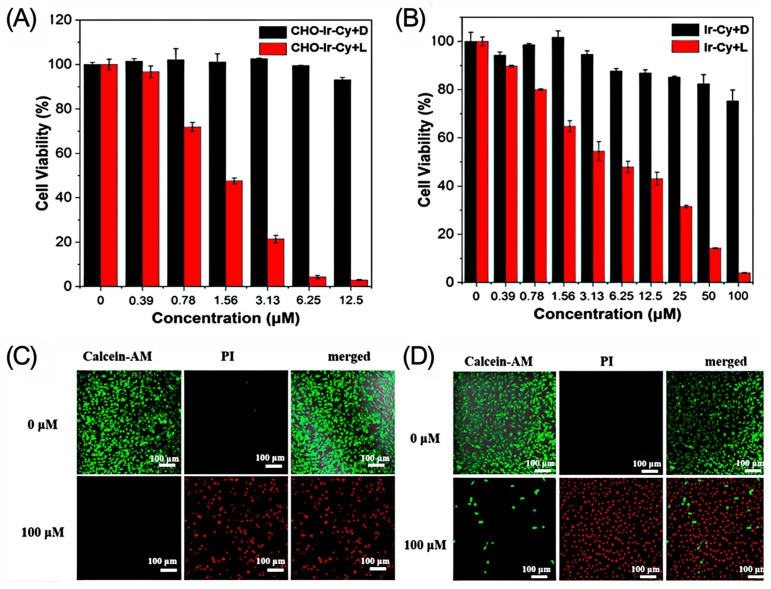
(**A**) **CHO−Ir−Cy**, (**B**) Ir−Cy pretreated with 4T1 cells in the absence of light and illumination; (**C**) Fluorescence image of 4T1 cells co-stained with Calcein-AM/PI after 3 min irradiation with an 808 nm (0.50 W cm^−2^) laser in the presence of **CHO−Ir−Cy** and (**D**) Ir−Cy.

We systematically studied the cytotoxicity of **CHO−Ir−Cy** and Ir−Cy using a 3-(4,5-dimethylthiazol-2-yl)-2,5-diphenyltetrazolium bromide (MTT) assay. Succinate dehydrogenase in living cells can reduce exogenous MTT to blue–purple formazan crystals and deposit it in cells, but dead cells do not exhibit this phenomenon. In a certain cell number range, the number of crystals formed by MTT is proportional to the number of cells. The number of living cells is judged according to the measured absorbance (OD value), and the OD value is directly proportional to the number of living cells. **CHO−Ir−Cy** and Ir−Cy with different concentration gradients (0–100 μM) were incubated with 4T1 cells. After 3 min irradiation with an 808 nm laser, with the increase in photosensitizer concentration gradient, the cell survival rate decreased obviously. Compared with Ir−Cy, when the concentration of **CHO−Ir−Cy** was 12.5 μM, the cell survival rate was as low as 10% (Figure 7A), but when the concentration of Ir−Cy was 100 μM, the cell survival rate could be as low as 10% (Figure 7B). This aligns with the earlier findings that **CHO−Ir−Cy** demonstrates superior ^1^O_2_ generation capacity in solution. This might also be due to the fact that the molar absorption coefficients of **CHO−Ir−Cy** and Ir−Cy at 808 nm are 100,019 M^−1^cm^−1^ and 38,584 M^−1^cm^−1^, respectively; there is a significant difference in their absorbance at the irradiation wavelengths. At the same time, compared with Ir−Cy, the survival rate of cells co-incubated with **CHO−Ir−Cy** in dark conditions did not decrease significantly. The experimental data of cell phototoxicity show that **CHO−Ir−Cy** has high biocompatibility, low dark toxicity and good phototoxicity, with great potential for clinical application in photodynamic cancer therapy.

### 2.7. Living/Dead Cell Staining Experiment

To visually demonstrate the PDT effect on cells, the viability of cells was monitored using a live/dead cell staining assay. In this assay, Calcein AM (green fluorescence) was used to stain live cells, and Propidium iodide (PI) (red fluorescence) was used to stain dead cells. Cells without a photosensitizer (0 μM) served as the control group. After irradiation, as shown in Figure 7C,D, the control group exhibited strong green fluorescence in the Calcein AM channel, while no significant red fluorescence was observed in the PI channel, indicating minimal cell death in the absence of a photosensitizer. Conversely, when treated with 100 μM photosensitizer, intense red fluorescence appeared in the PI channel, demonstrating substantial cell death under PDT. These results align with the MTT assay data, confirming that both **CHO−Ir−Cy** and Ir−Cy exert strong cytotoxic effects. Notably, in the Calcein AM/PI merged images of **CHO−Ir−Cy**-treated cells, virtually no green fluorescence was detected, further highlighting its superior cell-killing efficacy compared to Ir−Cy.

## 3. Materials and Methods

### 3.1. General Information

The material for organic synthesis, indocyanine green (ICG), was purchased from Anhui Zesheng Technology Co., LTD. Fetal bovine serum (FBS) and RPMI Medium 1640 were purchased from Beijing, China, Solaibao Technology Co., LTD. 3-(4,5-Dimethylthiazol-2-yl)-2,5-diphenyltetrazolium bromide (MTT), 2′,7′-Dichlorofluorescence diacetate (DCFH-DA) and the cell viability (live/dead cell staining) assay kit were purchased from Shanghai, China, Beyotime Biotechnology Co., Ltd.

### 3.2. Synthesis and Characterization of Complex

#### 3.2.1. Synthesis of Cy

**Figure 8:**
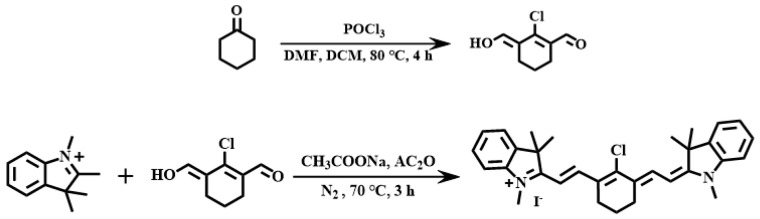
Synthesis steps for Cy.

Ligand Cy was synthesized in Figure 8 according to the literature method. At 0 °C, a uniformly mixed solution of phosphorus oxychloride (1.85 mL, 20 mmol) and ultra-dry dichloromethane (1.5 mL, 5 mmol) was added dropwise into a two-necked flask containing 10 mL ultra-dry dichloromethane and 10 mL ultra-dry N,N- dimethylformamide solution, and after 30 min, cyclohexanone (0.5 g, 5 mmol) was added dropwise, stirred and refluxed at 80 °C for 4 h and cooled with ice water overnight.

2-chloro-3-(hydroxymethylene)-cyclohexyl-1-ene formaldehyde (0.0863 g, 0.5 mmol), 1,2,3,3-tetramethyl-3H-indolium iodide (0.3 g, 1 mmol) and sodium acetate (0.0820 g, 1 mmol) synthesized in the previous step were mixed. The crude product was purified via silica gel column chromatography, with dichloromethane and ethyl acetate (10:5, V:V) as eluents, and a green solid was obtained with a yield of 70%.^1^H NMR (600 MHz, DMSO-d6, δ [ppm]): δ 8.25 (d, J = 14.2 Hz, 1H), 7.63 (d, J = 7.4 Hz, 1H), 7.50–7.38 (m, 2H), 7.33–7.26 (m, 1H), 6.31 (d, J = 14.2 Hz, 1H), 3.69 (s, 3H), 2.72 (t, J = 6.2 Hz, 2H), 1.90–1.82 (m, 1H), 1.68 (s, 6H).

#### 3.2.2. Preparation of Metal Iridium Complex Ir−Cy

Add ppy-Ir-H (0.0356 g, 0.05 mmol) and Cy (0.0733 g, 0.12 mmol) and NaH (0.0036 g, 0.15 mmol) into a round-bottomed flask, add 5 mL of ultra-dry N, N- dimethylformamide solution as a solvent, and react at room temperature for 48 h under the condition of avoiding light in a nitrogen atmosphere. After the reaction, the solvent was dried in vacuum, purified via silica gel column, and the blue solid was obtained using dichloromethane and methanol (20:1, V:V) as eluents, with a yield of 35%. ^1^H NMR (500 MHz, CDCl_3_, δ [ppm]) δ 8.71 (d, J = 8.9 Hz, 1H), 8.11 (d, J = 8.9 Hz, 1H), 7.76 (d, J = 8.2 Hz, 1H), 7.73 (d, J = 5.9 Hz, 1H), 7.66 (d, J = 14.2 Hz, 1H), 7.60–7.58 (m, 1H), 7.56–7.53 (m, 2H), 7.50–7.47 (m, 1H), 7.44 (dd, J = 7.9, 1.3 Hz, 1H), 7.41–7.38 (m, 1H), 7.33–7.28 (m, 2H), 7.26–7.22 (m, 3H), 7.10 (d, J = 7.5 Hz, 1H), 7.09–7.06 (m, 1H), 7.06–7.02 (m, 2H), 7.00 (dd, J = 8.0, 2.8 Hz, 2H), 6.95 (d, J = 7.5 Hz, 1H), 6.89–6.86 (m, 1H), 6.81–6.78 (m, 2H), 6.76 (dd, J = 7.4, 1.3 Hz, 1H), 6.71–6.68 (m, 1H), 6.65 (d, J = 3.5 Hz, 1H), 6.64–6.61 (m, 1H), 6.44 (d, J = 7.3, 5.8, 1.4 Hz, 1H), 6.27 (dd, J = 7.7, 1.1 Hz, 1H), 6.23 (dd, J = 7.6, 1.1 Hz, 1H), 6.13 (d, J = 5.2 Hz, 1H), 6.10 (d, J = 5.1 Hz, 1H), 3.58 (d, J = 5.2 Hz, 6H), 2.79 (td, J = 15.5, 7.4 Hz, 2H), 2.60 (s, 2H), 1.93 (t, J = 6.7 Hz, 2H), 1.15 (d, J = 21.7 Hz, 12H). ESI-MS: [m/z] = 580.2021 (M^2+^) (calcd: 580.21 (M^2+^).

#### 3.2.3. Preparation of Metal Iridium Complex CHO−Ir−Cy

**Figure 9:**
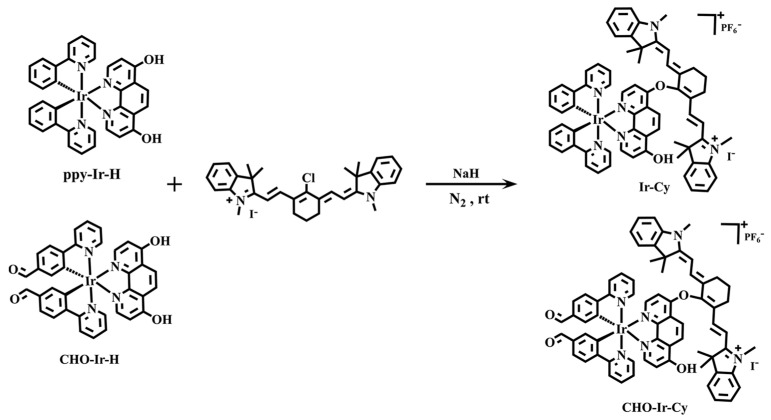
Synthesis steps for Ir−Cy and **CHO−Ir−Cy**.

The preparation of metallic iridium complex **CHO−Ir−Cy** in Figure 9 was similar to that of Ir-Cy. The metallic iridium complex ppy-Ir-H was replaced with CHO-Ir-H, and the blue target product was obtained with a yield of 33%. ^1^H NMR (500 MHz, CDCl_3_, δ [ppm]): δ 9.64 (s, 1H), 9.55 (s, 1H), 8.78 (d, J = 8.9 Hz, 1H), 8.16 (d, J = 8.9 Hz, 1H), 7.98 (d, J = 8.2 Hz, 1H), 7.87(d, 1H)7.78 (d, J = 8.1 Hz, 1H), 7.74 (d, J = 6.0 Hz, 1H), 7.72 (d, J = 8.0 Hz, 1H), 7.69 (d, J = 4.6 Hz, 2H), 7.66 (s, 1H), 7.53 (d, J = 7.9 Hz, 1H), 7.47 (s, 1H), 7.44 (d, J = 8.4 Hz, 2H), 7.35 (d, J = 6.1 Hz, 2H), 7.31–7.27 (m, 2H), 7.25 (s, 1H), 7.16 (t, J = 7.5 Hz, 1H), 7.10–7.06 (m, 3H), 7.03 (t, J = 7.4 Hz, 2H), 6.87–6.82 (m, 2H), 6.75 (d, J = 1.5 Hz, 1H), 6.71 (d, J = 1.6 Hz, 1H), 6.67 (t, J = 6.7 Hz, 1H), 6.56 (d, J = 6.8 Hz, 1H), 6.18 (d, J = 14.3 Hz, 1H), 6.06 (d, J = 14.1 Hz, 1H), 3.64 (s, 3H), 3.58 (s, 4H), 2.89–2.72 (m, 3H), 2.61 (s, 3H), 1.96–1.91 (m, 2H), 1.25 (s, 3H), 1.18 (s, 6H). ESI-MS: [*m*/*z*] = 580.2021 (M^2+^) (calcd: 608.21 (M^2+^).

## 4. Conclusions

In this study, we designed and synthesized a metal iridium complex photosensitizer with NIR light absorption and emission performance through a reasonable molecular design strategy and successfully applied it to photodynamic cancer therapy. We synergistically combined the strengths of cyanine (e.g., tunable long-wavelength excitation) and transition metal complexes (e.g., high ^1^O_2_ quantum yield), thereby addressing the inherent limitation of short-wavelength absorption in conventional iridium-based photosensitizers and achieving the construction of a long-wavelength excitable transition metal photosensitizer, namely **CHO−Ir−Cy**. The optimized photosensitizer, **CHO−Ir−Cy**, demonstrated exceptional ^1^O_2_ generation efficiency and was rapidly internalized by 4T1 cancer cells, exhibiting excellent biocompatibility, strong phototoxicity under NIR irradiation and negligible dark toxicity. This study successfully validates the therapeutic potential of NIR-activated iridium complexes in PDT, highlighting their future clinical applicability. Our work establishes a novel molecular design paradigm for developing high-performance transition metal photosensitizers with NIR absorption/emission capabilities, offering a strategic pathway to advance the clinical translation of PDT in oncology.

## Data Availability

All data generated or analyzed during this study are included in this published article and its Supplementary Information files.

## Supplementary Materials

The following supporting information can be downloaded at: https://www.mdpi.com/article/10.3390/molecules30122662/s1, Figure S1: ^1^H NMR spectrum of Cy in DMSO-*d*_6_.; Figure S2: Mass spectrum of Cy; Figure S3: ^1^H NMR spectrum of Ir-Cy; Figure S4: Mass spectrum of Ir-Cy; Figure S5: ^1^H NMR spectrum of **CHO-Ir-Cy**.; Figure S6: Mass spectrum of **CHO-Ir-Cy**.; Figure S7: (A) UV-Vis absorption spectrum of DPBF in the presence of **CHO-Ir-Cy** and (B) Ir-Cy (10 μM) without illumination.; Figure S8: UV-Vis absorption spectrum of DPBF under illumination.; Figure S9: The absorption spectra under several different computational functionals.; Table S1: Photophysical data of CHO-Ir-Cy and Ir-Cy; Table S2: Excitation energies E (eV), vibronic strengths f and major leptonic components of material molecules calculated at the TD-B3LYP/6-31G(d) level; Table S3: Individual energy difference data for CHO-Ir-Cy, Ir-Cy and Cy; Table S4: SOC values(ξ) for CHO-Ir-Cy, Ir-Cy and Cy; Table S5: CHO-Ir-Cy structural coordinate values.; Table S6: Ir-Cy structural coordinate values.; Table S7: Cy structural coordinate values.; Table S8: Complex excitation energy information.

## References

[1] Kaiser A.M., Gatto A., Hanson K.J., Zhao R.L., Raj N., Ozawa M.G., Seoane J.A., Bieging-Rolett K.T., Wang M., Li I. (2023). p53 governs an AT1 differentiation programme in lung cancer suppression. Nature.

[2] Lewnard J.A., Kang G., Laxminarayan R. (2023). Attributed causes ofexcess mortality during theCOVID-19pandemic ina south Indian city. Nat. Commun..

[3] Mani K., Deng D., Lin C., Wang M., Hsu M.L., Zaorsky N.G. (2024). Causes ofdeath among people living with metastatic cancer. Nat. Commun..

[4] Siegel R.L., Miller K.D., Fuchs H.E., Jemal A. (2022). Cancer statistics, 2022. CA Cancer J. Clin..

[5] Zhang X., Lyu Y., Li J., Yang X., Lan Z., Chen Z. (2024). Bimetallic Nanozymes-Integrated Parachute-Like Au_2_Pt @PMO@ICG Janus Nanomotor with Dual Propulsion for Enhanced Tumor Penetration and Synergistic PTT/PDT/CDT Cancer Therapy. Adv. Funct. Mater..

[6] Yu H., Wang Y., Chen Y., Cui M., Yang F., Wang P., Ji M. (2023). Transmissible H-aggregated NIR-II fluorophore to the tumor cell membrane for enhanced PTT and synergistic therapy of cancer. Nano Converg..

[7] Bian X., Piipponen M., Liu Z., Luo L., Geara J., Chen Y., Sangsuwan T., Maselli M., Diaz C., Bain C.A. (2024). Epigenetic memory of radiotherapy in dermal fibroblasts impairs wound repair capacity in cancer survivors. Nat. Commun..

[8] Jenniffer L., Anna S.-A., Jordi B.-R., Alba R.-B., Ana M., Noemí M.-R., Daniele L.R., Elisa I.R., Marc G., Melissa Z. (2023). Long-term platinum-based drug accumulation in cancer-associated fibroblasts promotes colorectal cancer progression and resistance to therapy. Nat. Commun..

[9] Zhou L., Lyu J., Liu F., Su Y., Feng L., Zhang X. (2023). Immunogenic PANoptosis-Initiated Cancer Sono-Immune Reediting Nanotherapy by Iteratively Boosting Cancer Immunity Cycle. Adv. Mater..

[10] Ke L., Wei F., Xie L., Karges J., Chen Y., Ji L., Chao H. (2022). A Biodegradable Iridium(III) Coordination Polymer for Enhanced Two-Photon Photodynamic Therapy Using an Apoptosis-Ferroptosis Hybrid Pathway. Angew. Chem. Int. Ed. Engl..

[11] Zhao X., Liu J., Fan J., Chao H., Peng X. (2021). Recent progress in photosensitizers for overcoming the challenges of photodynamic therapy: From molecular design to application. Chem. Soc. Rev..

[12] Fang L., Huang R., Gong W., Ji Y., Sun Y., Gou S., Zhao J. (2023). A Self-Assembly-Induced Exciton Delocalization Strategy for Converting a Perylene Diimide Derivative from a Type-II to Type-I Photosensitizer. Small.

[13] Lu B., Xia J., Quan H., Huang Y., Zhang Z., Zhan X. (2023). End Group Engineering for Constructing A−D−A Fused-Ring Photosensitizers with Balanced Phototheranostics Performance. Small.

[14] Sun F., Chen Y., Lam K.W.K., Du W., Liu Q., Han F., Li D., Lam J.W.Y., Sun J., Kwok R.T.K. (2024). Glutathione-responsive Aggregation-induced Emission Photosensitizers for Enhanced Photodynamic Therapy of Lung Cancer. Small.

[15] Jiao Q., Zheng Y., Xie Q., Luo X., Zhou S., Pei S., Zhang T., Wu X., Xu K., Zhong W. (2023). A Dual-Responsive Morphologically-Adaptable Nanoplatform for Targeted Delivery of Activatable Photosensitizers in Precision Photodynamic Therapy. Small.

[16] Cheng H.B., Qiao B., Li H., Cao J., Swamy K.M.K., Zhao J., Wang Z., Lee J., Liang X.J., Yoon J. (2021). Protein-Activatable Diarylethene Monomer as a Smart Trigger of Noninvasive Control Over Reversible Generation of Singlet Oxygen: A Facile, Switchable, Theranostic Strategy for Photodynamic-Immunotherapy. J. Am. Chem. Soc..

[17] Lu H., Jiang X., Chen Y., Peng K., Huang Y., Zhao H., Chen Q., Lv F., Liu L., Wang S. (2020). Cyclometalated iridium(iii) complex nanoparticles for mitochondria-targeted photodynamic therapy. Nanoscale.

[18] Yang J., Fang H.J., Cao Q., Mao Z.W. (2021). The design of cyclometalated iridium(iii)-metformin complexes for hypoxic cancer treatment. Chem. Commun..

[19] Lu G., Wu Z.G., Wu R., Cao X., Zhou L., Zheng Y.X., Yang C. (2021). Semitransparent Circularly Polarized Phosphorescent Organic Light-Emitting Diodes with External Quantum Efficiency over 30% and Dissymmetry Factor Close to 10−2. Adv. Funct. Mater..

[20] Liu S., Han J., Wang W., Chang Y., Wang R., Wang Z., Li G., Zhu D., Martin R.B. (2022). AIE-active Ir(III) complexes functionalised with a cationic Schiff base ligand: Synthesis, photophysical properties and applications in photodynamic therapy. Dalton Trans..

[21] Liu S., Chen H., Wu Q., Sun Y., Pei Y., Wang Z., Zhu D., Li G., Bryce M.R., Chang Y. (2024). Self-Chemiluminescence-Trig gered Ir(III) Complex Photosensitizer for Photodynamic Therapy against Hypoxic Tumor. Inorg. Chem..

[22] Huang S., Li Y., Xie X., Tong J., Shan G.-G., Qin C., Xiao X., Wang Q., Li Y., Wang H. (2025). Enhanced ROS generation in AIE-active iridium(III) photosensitizers by cationization engineering for advanced photodynamic therapy. Inorg. Chem. Front..

[23] Wen L.L., Zhang J.M., Han Y.P., Duan Y.C., Xie W.F., Shao K.Z., Shan G.G., Su Z.M. (2023). Boosting the efficiency of deep-red Ir(iii) complexes by modulating nitrogen atoms for high-performance OLEDs. Inorg. Chem. Front..

[24] Li X., Zang C.-X., Gao Y., Wen L.-L., Shao K.-Z., Ding G.-Y., Shan G.-G., Xie W.-F., Su Z.-M. (2022). Novel Ir(III) Complexes with NHC-Based Ancillary Ligands for Efficient Nondoped OLEDs. Inorg. Chem..

[25] Tong J., Liu A., Huang S., Zhou D., Gao Y., Wang Y., Shan G.G. (2023). Precise ligand engineering of Ir(III)-based photo sensitizer with aggregation-induced emission for image-guided photodynamic therapy. Luminescence.

[26] Song W.L., Mao H.T., Gao Y., Yao Y.X., Shan G.G., Su Z.M. (2024). Understanding AIE and ACQ phenomenon of organ ometallic iridium(III) complexes by simple cationization engineering. Chin. Chem. Lett..

[27] Jiang Y., Li G., Che W., Liu Y., Xu B., Shan G., Zhu D., Su Z., Bryce M.R. (2017). A neutral dinuclear Ir(III) complex for anti-counterfeiting and data encryption. Chem. Commun..

[28] Song W., Gao J., Gao Y., Shan G.-G., Geng Y., Shao K., Su Z.-M. (2024). Constructing anion–π interactions in cationic iridium(III) complexes to achieve aggregationinduced emission properties. Inorg. Chem. Front..

[29] Li G., Ren X., Shan G., Che W., Zhu D., Yan L., Su Z., Bryce M.R. (2015). New AIE-active dinuclear Ir(III) complexes with reversible piezochromic phosphorescence behaviour. Chem. Commun..

[30] Liu B., Monro S., Li Z., Jabed M.A., Ramirez D., Cameron C.G., Colón K., Roque J., Kilina S., Tian J. (2019). A New Class of Homoleptic and Heteroleptic Bis(terpyridine) Iridium(III) Complexes with Strong Photodynamic Therapy Effects. ACS Appl. Bio Mater..

[31] Karges J., Heinemann F., Jakubaszek M., Maschietto F., Subecz C., Dotou M., Vinck R., Blacque O., Tharaud M., Goud B. (2020). Rationally designed long-wavelength absorbing Ru(II) polypyridyl complexes as photosensitizers for photodynamic therapy. J. Am. Chem. Soc..

[32] Wang Z., Li L., Wang W., Wang R., Li G., Bian H., Zhu D., Bryce M.R. (2023). Self-assembled nanoparticles based on cationic mono-/AIE tetra-nuclear Ir(III) complexes: Long wavelength absorption/near-infrared emission photosensitizers for photodynamic therapy†. Dalton Trans..

[33] Zhu W., Liu S., Wang Z., Shi C., Zhang Q., Wu Z., Li G., Zhu D. (2023). An AIE Metal Iridium Complex: Photophysical Properties and Singlet Oxygen Generation Capacity. Molecules.

[34] Zhang L., Che W., Yang Z., Liu X., Liu S., Xie Z., Zhu D., Su Z., Tang B.Z., Bryce M.R. (2020). Bright red aggregation-induced emission nanoparticles for multifunctional applications in cancer therapy. Chem. Sci..

[35] Pei Y., Sun Y., Zhu D. (2024). Phosphorescent Sensor Based on Iridium(III) Complex with Aggregation-Induced Emission Activity for Facile Detection of Volatile Acids. Molecules.

[36] Liu N., He S., Cheng Z., Hu J. (2025). Enhancing the fluorescence emission of the NIR-II fluorophores: Strategies, mechanisms, challenges, and opportunities. Coord. Chem. Rev..

[37] Wilson J.S., Chawdhury N., Al-Mandhary M.R., Younus M., Khan M.S., Raithby P.R., Kohler A., Friend R.H. (2001). The energy gap law for triplet states in Pt-containing conjugated polymers and monomers. J. Am. Chem. Soc..

[38] Wang S.F., Su B.K., Wang X.Q., Wei Y.C., Kuo K.H., Wang C.H., Liu S.H., Liao L.S., Hung W.Y., Fu L.W. (2022). Polyatomic molecules with emission quantum yields > 20% enable efficient organic light-emitting diodes in the NIR (II) window. Nat. Photonics.

[39] Boyde S., Strouse G.F., Jones W.E., Meyer T.J. (1990). The effect on MLCT excited states of electronic delocalization in the acceptor ligand. J. Am. Chem. Soc..

[40] Zhao J., Gao Y., Huang R., Chi C., Sun Y., Xu G., Xia X.H., Gou S. (2023). Design of Near-Infrared-Triggered metallo-photosensitizers via a self-assembly-induced vibronic decoupling strategy. J. Am. Chem. Soc..

[41] Treadway J.A., Loeb B., Lopez R., Anderson P.A., Keene F.R., Meyer T.J. (1996). Effect of delocalization and rigidity in the acceptor ligand on MLCT excited-state decay. Inorg. Chem..

[42] Liu S., Wang Z., Wu Z., Chen H., Zhu D., Li G., Yan M., Martin R.B., Chang Y. (2024). Long-wavelength triggered iridium(III) complex nanoparticles for photodynamic therapy against hypoxic cancer. Chem. Commun..

[43] Zhao J., Yan K., Xu G., Liu X., Zhao Q., Xu C., Gou S. (2021). An Iridium (III) Complex Bearing a Donor–Acceptor–Donor Type Ligand for NIR-Triggered Dual Phototherapy. Adv. Funct. Mater..

[44] Liu B., Jiao J., Xu W., Zhang M., Cui P., Guo Z., Deng Y., Chen H., Sun W. (2021). Highly Efficient Far-Red/NIR-Absorbing Neutral Ir(III) Complex Micelles for Potent Photodynamic/Photothermal Therapy. Adv. Mater..

[45] Lv F., Feng E., Lv S., Liu D., Song F. (2023). Metal-Coordination-Mediated H-Aggregates of Cyanine Dyes for Effective Photothermal Therapy. Chem.—A Eur. J..

[46] Wang W., Wang L., Liu S., Xie Z. (2017). Metal−Organic Frameworks@Polymer Composites Containing Cyanines for Near-Infrared Fluorescence Imaging and Photothermal Tumor Therapy. Bioconjug. Chem..

[47] Zhao X.B., Ha W., Gao K., Shi Y.P. (2020). Precisely Traceable Drug Delivery of Azoreductase-Responsive Prodrug for Colon Targeting via Multimodal Imaging. Anal. Chem..

[48] Guo Z.Y., Li C.X., Gao M., Han X., Zhang Y.-J., Zhang W.-J., Li W.-W. (2021). Inside Cover: Mn−O Covalency Governs the Intrinsic Activity of Co-Mn Spinel Oxides for Boosted Peroxymonosulfate Activation. Angew. Chem. Int. Ed..

[49] Zheng Y., Lu H., Jiang Z., Guan Y., Zou J., Wang X., Cheng R., Gao H. (2017). Low-power white light triggered AIE polymer nanoparticles with high ROS quantum yield for mitochondria-targeted and image-guided photodynamic therapy. J. Mater. Chem. B.

