# Supplementary material for: Constructing 1 + 1 > 2 Photosensitizers Based on NIR Cyanine–Iridium(III) Complexes for Enhanced Photodynamic Cancer Therapy
Source: Molecules. 2025 Jun 19;30(12):2662. doi: 10.3390/molecules30122662 (PMC12196380; doi:10.3390/molecules30122662)
Supplement: Supplementary file 1 [file molecules-30-02662-s001.zip › molecules-3670584-supplementary.pdf]

Supporting information

Constructing 1 + 1 > 2 Photosensitizers Based on NIR Cyanine-–Iridium(III) Complexes for Enhanced Photodynamic Cancer Therapy

Ziwei Wang [1], Weijin Wang [1], Qi Wu [1], Dongxia Zhu [1,*]

1 Key Laboratory of Nanobiosensing and Nanobioanalysis at Universities of Jilin Province, Department of Chemistry, Northeast Normal University, 5268 Renmin Street, Changchun, Jilin Province 130024, P. R. China.; zhudx047@nenu.edu.cn

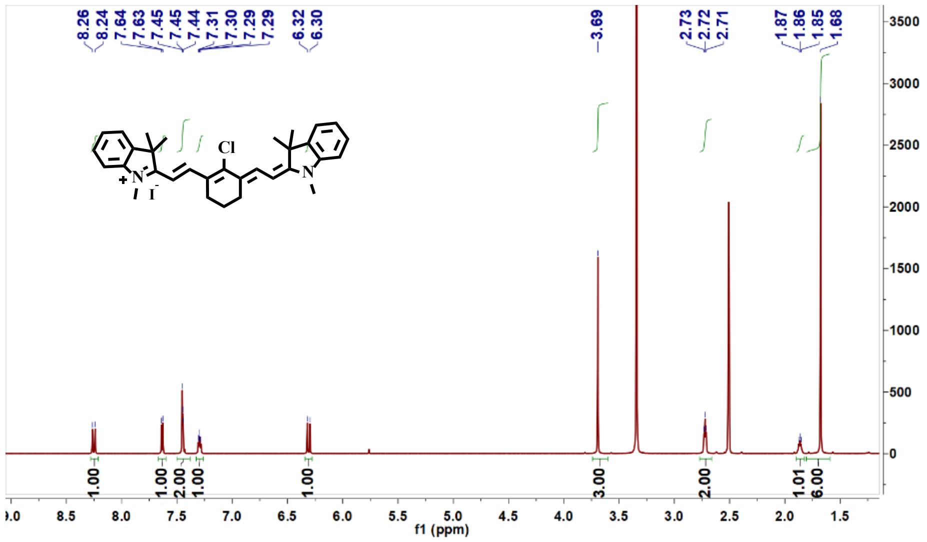

Figure S1. ^{1}H NMR spectrum of Cy in DMSO-d_6.

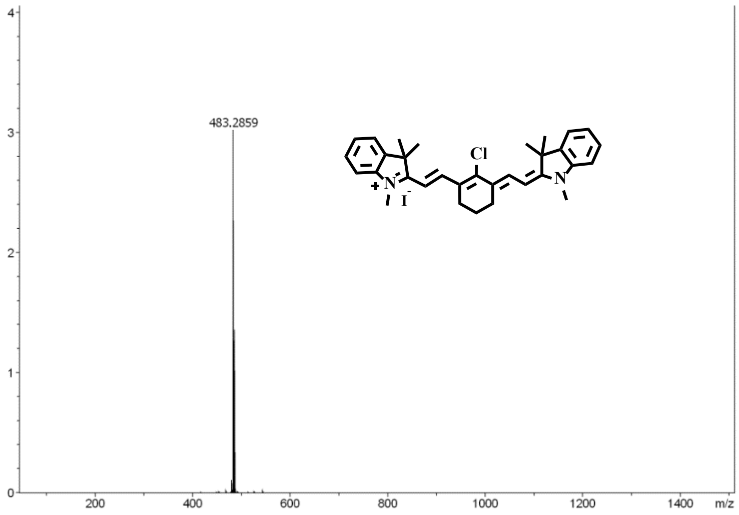

Figure S2. Mass spectrum of Cy.

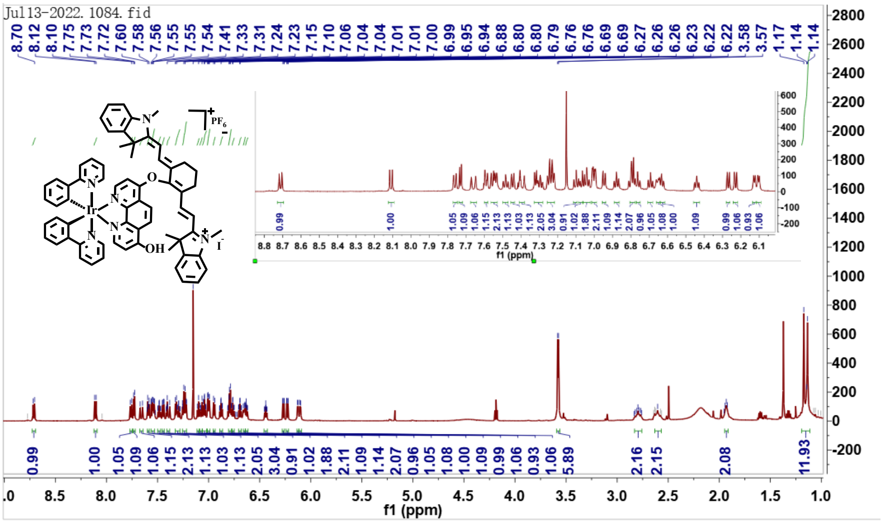

Figure S3. ^{1}H NMR spectrum of Ir-Cy.

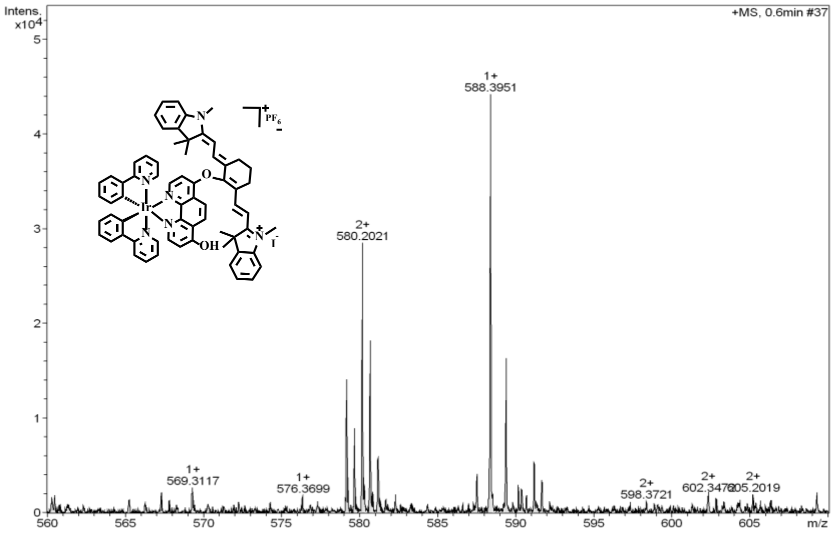

Meas. m/z	#	Ion Formula	m/z	err [ppm]	mSigma	# Sigma	Score	rdb	e⁻ Conf	N-Rule
580.2021	1	C66H59IrN6O2	580.2162	24.3	33.1	1	100.00	41.0	even	ok

Figure S4. Mass spectrum of Ir-Cy**.**

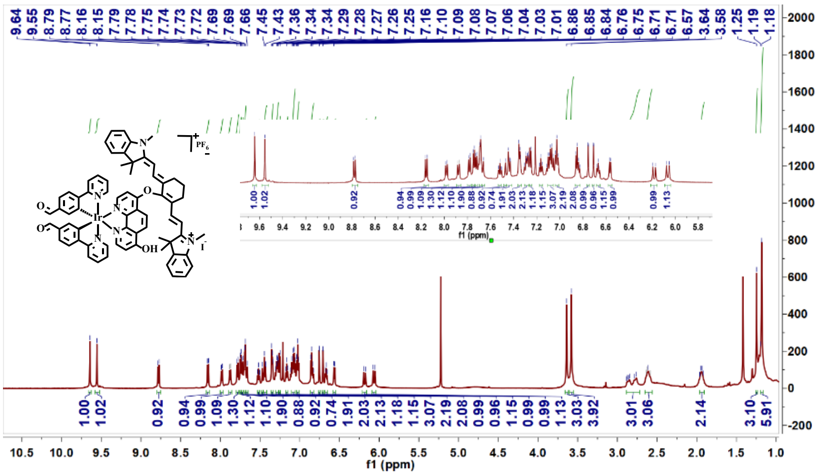

Figure S5. ^{1}H NMR spectrum of **CHO-Ir-Cy**.

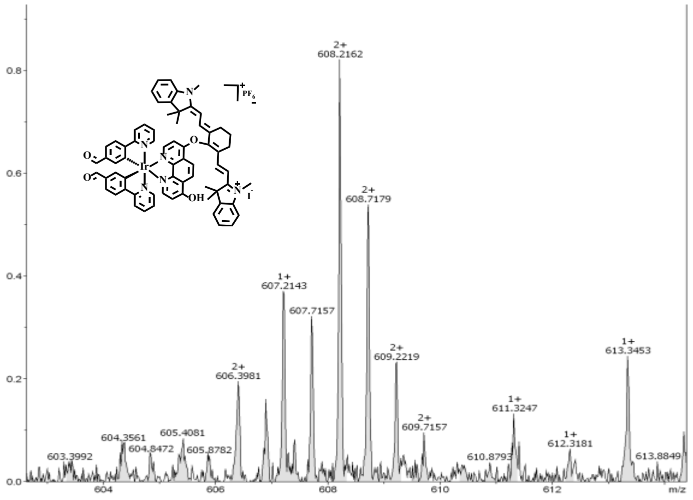

Figure S6. Mass spectrum of **CHO-Ir-Cy.**

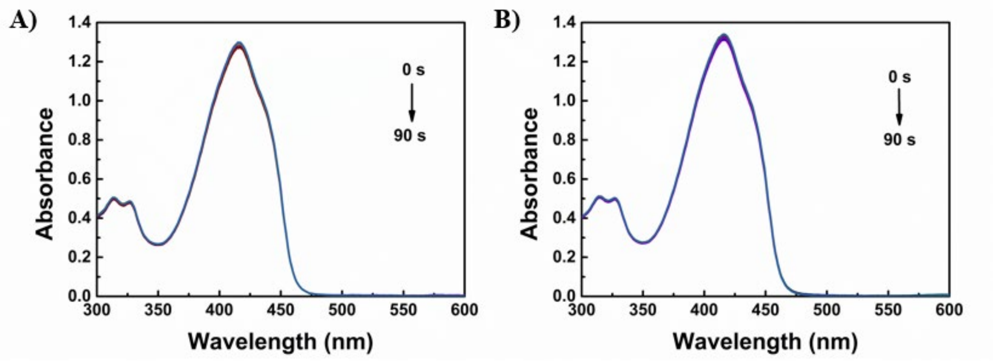

Figure S7. A) UV-Vis absorption spectrum of DPBF in the presence of **CHO-Ir-Cy** and B) Ir-Cy (10 μM) without illumination.

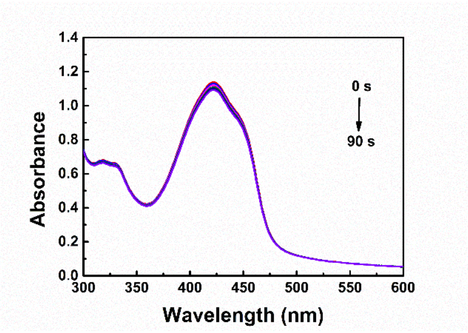

Figure S8. UV-Vis absorption spectrum of DPBF under illumination.

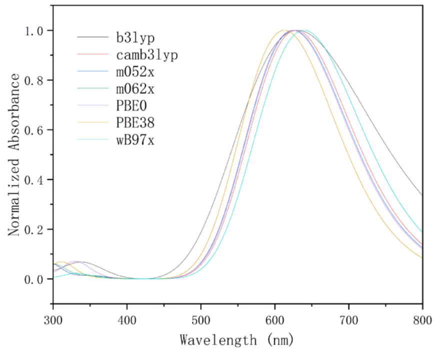

Figure S9. The absorption spectra under several different computational functionals.

Table S1. Photophysical data of **CHO-Ir-Cy** and **Ir-Cy**.

	λ_{abs} (nm)	λ_{em} (nm)	Φ_P (%)	τ_P② (ns)	ε (M^{-1} cm^{-1})
**CHO-Ir-Cy**①	300；380；790	800	7.71	4.38	77255；29409；199140；
**Ir-Cy**①	260；370；790	800	1.6	4.12	49326；14906；78021；

①Measured in DMSO, the concentration is 1.0×10^{-5} M, λ_{ex} = 730 nm; ② It was determined in normal oxygen environment without oxygen removal test.

Table S2. Excitation energies E (eV), vibronic strengths f and major leptonic components of material molecules calculated at the TD-B3LYP/6-31G(d) level

molecular	parameterisation	stimulus	amplitude	Wavelength/nm
**CHO-Ir-Cy**	S_2	2.1216	1.92310	584.39
**Ir-Cy**	S_3	2.1277	1.88740	582.71

**Cy**	S_1	2.1682	2.20630	571.83

Table S3 Individual energy difference data for **CHO-Ir-Cy**, Ir-Cy and Cy

	Ir-Cy			CHO-Ir-Cy			Cy		
n	Sn(eV)	Tn(eV)	ΔE_{S1-Tn}	Sn(eV)	Tn(eV)	ΔE_{S1-Tn}	Sn(eV)	Tn(eV)	ΔE_{S1-Tn}
1	1.98	1.01	0.97	1.97	0.96	1.00	1.96	0.95	1.01
2	2.34	2.14	0.16	2.43	2.17	0.20	2.98	2.13	0.16
3	2.41	2.34	0.36	2.47	2.41	0.44	3.53	2.64	0.67
4	2.61	2.40	0.41	2.49	2.43	0.46	3.63	2.85	0.89
5	2.81	2.58	0.59	2.55	2.46	0.49	3.66	3.37	1.40
6	2.85	2.64	0.66	2.63	2.46	0.49	3.66	3.51	1.54
7	2.97	2.72	0.73	2.83	2.49	0.52	4.06	3.51	1.55
8	3.01	2.75	0.76	2.93	2.55	0.57	4.15	3.65	1.68
9	3.01	2.79	0.81	2.94	2.60	0.63	4.19	3.83	1.87
10	3.01	2.81	0.83	3.00	2.63	0.65	4.50	3.91	1.94
11	3.08	2.84	0.86	3.03	2.79	0.82	4.71	3.92	1.96
12	3.11	2.89	0.91	3.15	2.84	0.87	4.73	4.06	2.09
13	3.12	2.95	0.97	3.18	2.94	0.97	4.81	4.28	2.31
14	3.25	3.01	1.03	3.25	2.95	0.97	4.82	4.39	2.42
15	3.33	3.01	1.03	3.36	2.95	0.98	5.00	4.46	2.50

16	3.42	3.05	1.07	3.38	3.01	1.04	5.03	4.47	2.50
17	3.46	3.08	1.10	3.40	3.02	1.05	5.07	4.63	2.67
18	3.48	3.11	1.13	3.477	3.07	1.10	5.22	4.71	2.74
19	3.53	3.21	1.23	3.504	3.07	1.10	5.24	4.75	2.78
20	3.56	3.22	1.23	3.518	3.15	1.18	5.27	4.80	2.84
21	3.57	3.32	1.34	3.52	3.17	1.20	5.35	4.87	2.90
22	3.59	3.33	1.35	3.54	3.25	1.28	5.41	4.88	2.91
23	3.62	3.39	1.41	3.57	3.30	1.33	5.50	4.95	2.99
24	3.63	3.46	1.48	3.58	3.31	1.34	5.50	5.14	3.17
25	3.67	3.50	1.52	3.60	3.38	1.41	5.56	5.17	3.21
26	3.68	3.53	1.54	3.61	3.40	1.43	5.58	5.18	3.21
27	3.69	3.54	1.56	3.64	3.42	1.44	5.71	5.19	3.22
28	3.72	3.55	1.57	3.66	3.46	1.48	5.77	5.28	3.31
29	3.73	3.55	1.57	3.67	3.46	1.49	5.81	5.34	3.38
30	3.76	3.56	1.58	3.68	3.51	1.54	5.82	5.38	3.42

Table S4. SOC values（ξ） for **CHO-Ir-Cy**, Ir-Cy and Cy

	Cy	CHO-Ir-Cy	Ir-Cy
n	$\xi_{(S1\text{-}Tn)}$	$\xi_{(S1\text{-}Tn)}$	$\xi_{(S1\text{-}Tn)}$
1	0.02	9.18	8.52

Table S5 CHO-Ir-Cy structural coordinate values.

atom	X	Y	Z
C	13.19236	-0.044226	2.776623
H	12.639724	0.274332	3.654642
C	15.147162	-1.003998	1.762432
C	14.532382	-0.821789	0.487598
C	13.239897	-0.243451	0.469632
C	12.570727	-0.042557	-0.791487
C	13.225668	-0.411424	-1.988446
C	12.530157	-0.183965	-3.212694
C	11.260716	0.378147	-3.172198
H	10.69909	0.569073	-4.081963
C	10.696621	0.70199	-1.932935
H	9.704878	1.138687	-1.874764
C	10.062864	1.282215	3.204113
C	10.258893	2.424487	3.992434
H	10.682207	3.325843	3.554942
C	9.919445	2.444922	5.351062
C	9.357046	1.312077	5.961183
H	9.100653	1.355799	7.014595
C	9.136934	0.17225	5.205185
H	8.695284	-0.699443	5.6795
C	9.483741	0.150943	3.839844
C	9.281214	-1.014349	2.979114
C	8.669349	-2.218477	3.358165
H	8.303299	-2.335357	4.37121
C	8.515287	-3.246258	2.437563
H	8.030064	-4.17277	2.729106
C	8.982474	-3.066267	1.13225
H	8.874789	-3.83734	0.377363
C	9.582874	-1.859018	0.806391
H	9.950575	-1.660533	-0.192896
C	12.419328	3.434659	1.140318
H	13.140716	2.705171	1.487871
C	12.770751	4.759292	0.928337
H	13.789623	5.084098	1.108142
C	10.18812	3.828275	0.516005
C	8.872502	3.211181	0.353572
C	6.428307	1.907842	0.105923
C	7.552538	1.209767	0.564354
H	7.433247	0.160462	0.823924
N	11.316274	0.499907	-0.769323
N	9.731514	-0.857427	1.695905

I	10.555514	1.006971	1.263954
C	14.466574	-0.612721	2.906143
H	14.901766	-0.737145	3.889488
N	11.16859	2.973233	0.942588
C	8.800919	1.833491	0.693503
C	11.784431	5.648354	0.490681
H	12.0184	6.694272	0.317455
C	6.523577	3.267091	-0.232581
H	5.636632	3.784333	-0.583387
C	10.495032	5.177784	0.28508
H	9.715703	5.853195	-0.046785
C	7.741542	3.914698	-0.105909
H	7.809434	4.967454	-0.364226
C	15.163319	-1.1909	-0.743337
H	16.152866	-1.631828	-0.708682
C	14.533646	-0.989103	-1.938752
H	15.01337	-1.265085	-2.870771
N	12.586591	0.145687	1.605036
O	16.384116	-1.561875	1.750263
O	13.165549	-0.537345	-4.34181
H	12.620205	-0.34493	-5.123615
C	14.452743	-9.936263	2.726915
C	14.70632	-8.583718	2.515229
C	14.293838	-7.91678	1.358795
C	13.603099	-8.607288	0.372081
C	13.334539	-9.969029	0.562653
C	13.755797	-10.620605	1.725856
H	14.77587	-10.45911	3.62051
H	13.276529	-8.109183	-0.536855
H	12.797944	-10.524092	-0.200236
H	13.543113	-11.676744	1.858336
C	14.730013	-6.462285	1.431187
C	15.704537	-6.136558	0.272238
H	15.195953	-6.290384	-0.684957
H	16.051932	-5.100136	0.31086
H	16.57969	-6.792544	0.29715
C	13.49494	-5.528512	1.406422
H	13.779389	-4.474613	1.479123
H	12.952507	-5.670225	0.465917
C	15.440378	-6.426423	2.794318
H	12.811161	-5.757985	2.229455
C	16.08274	-5.360803	3.434055
H	16.552383	-5.585224	4.384868
C	16.203024	-4.054447	2.950123

H	15.746476	-3.813883	1.997947
C	16.925214	-3.026999	3.577868
C	17.074138	-1.769672	2.962982
C	17.944724	-0.747713	3.387616
C	21.380788	5.494776	1.438693
C	20.535052	4.38882	1.429256
C	19.707815	4.08858	0.343952
C	19.711172	4.909503	-0.775695
C	20.55267	6.029559	-0.789334
C	21.374769	6.314231	0.30525
H	22.028684	5.728493	2.276479
H	19.078808	4.692308	-1.632374
H	20.569391	6.680093	-1.657974
H	22.023735	7.183932	0.279216
N	20.342358	3.404035	2.429815
C	18.922296	2.821713	0.642552
C	17.40292	3.120766	0.649099
H	17.099649	3.479942	-0.339784
H	16.81414	2.228429	0.88203
H	17.159451	3.896752	1.381744
C	19.273014	1.717781	-0.385472
H	20.345846	1.503792	-0.382671
H	18.73735	0.786118	-0.181859
H	18.999957	2.055898	-1.39029
C	19.436583	2.470112	2.048449
C	19.106031	1.387859	2.870643
H	19.63463	1.324358	3.814857
C	18.190823	0.371579	2.577186
H	17.656866	0.415141	1.635827
C	21.047963	3.395317	3.707316
H	21.660457	4.291182	3.786841
H	20.332817	3.38822	4.535429
H	21.697561	2.517089	3.781259
C	15.958734	-8.009212	4.64633
H	15.545829	-7.366756	5.429949
H	15.715571	-9.043196	4.882509
H	17.047903	-7.900164	4.624169
C	18.681701	-0.954585	4.699149
H	18.789123	0.004482	5.218276
H	19.702549	-1.309952	4.494363
C	17.658438	-3.25741	4.887494
H	18.594851	-3.801446	4.693756
H	17.05813	-3.902002	5.539262
C	17.962061	-1.946117	5.617497

H	18.57086	-2.143784	6.505367
H	17.022664	-1.500106	5.970105
N	15.383219	-7.660987	3.351105
C	10.163292	3.672772	6.152147
H	10.567487	4.53389	5.574929
O	9.955611	3.762159	7.344576
C	5.125834	1.202553	-0.024019
H	5.138421	0.136338	0.293767
O	4.110702	1.718178	-0.44323

Table S6 Ir-Cy structural coordinate values.

atom	X	Y	Z
C	12.996251	0.08965	2.563784
H	12.342544	0.602636	3.261696
C	14.952945	-1.244167	2.093235
C	14.618848	-1.199863	0.703018
C	13.442491	-0.468957	0.359996
C	13.020004	-0.393483	1.020631
C	13.790429	-1.025411	2.020108
C	13.344447	-0.912888	3.369096
C	12.182267	-0.202003	3.635964
H	11.807944	-0.091032	4.649458
C	11.479072	0.376075	2.572984
H	10.562471	0.929207	2.751087
C	10.080777	1.831265	2.214554
C	10.28308	3.069649	2.842361
H	10.880547	3.834696	2.355139
C	9.716166	3.35303	4.087651
H	9.884067	4.324068	4.546792
C	8.925353	2.403886	4.744381
H	8.482611	2.629881	5.709544
C	8.701763	1.169674	4.145618
H	8.08232	0.437994	4.656868
C	9.269017	0.881179	2.891847
C	9.070489	-0.385751	2.191855
C	8.265996	-1.450519	2.627949
H	7.730897	-1.364126	3.566171
C	8.140502	-2.596727	1.856208
H	7.507861	-3.413556	2.190392
C	8.828861	-2.679915	0.641385
H	8.752249	-3.551691	0.000996
C	9.61345	-1.604357	0.254782
H	10.157452	-1.607908	0.681898
C	13.008343	3.356323	0.306203
H	13.56475	2.624258	0.879119

C	13.560663	4.579358	0.040116
H	14.574405	4.821202	0.259793
C	10.970603	3.867621	0.749726
C	9.630682	3.370922	1.047909
C	7.110321	2.294988	1.524839
H	6.124067	1.873919	1.702603
C	8.04692	1.570699	-0.78283
H	7.76213	0.597425	0.394858
N	11.868708	0.287548	1.300575
N	9.737285	-0.488121	1.000581
I	10.84852	1.189468	0.458348
C	14.122455	-0.605125	3.007173
H	14.358584	-0.653765	4.06433
N	11.753115	3.001705	0.033597
C	9.327802	2.083198	0.528826
C	12.778666	5.479008	0.772325
H	13.173165	6.448781	1.060173
C	7.426087	3.559265	2.034078
H	6.693982	4.119851	2.606988
C	11.486642	5.118749	1.124733
H	10.864166	5.804665	1.686755
C	8.685125	4.096257	1.794607
H	8.924657	5.079782	2.188982
C	15.353083	-1.846397	0.347681
H	16.2296	-2.427339	0.101321
C	14.960826	-1.756366	1.651526
H	15.529614	-2.25021	2.430897
N	12.661101	0.171648	1.276374
O	15.981807	-1.933432	2.664124
O	14.095113	-1.517601	4.305854
H	13.715482	-1.386894	5.191364
C	14.679294	-10.416489	2.975173
C	14.817585	-9.031868	2.931534
C	13.839928	-8.165084	3.426071
C	12.678742	-8.680961	3.985665
C	12.515849	-10.07127	4.040673
C	13.505224	-10.923777	3.54108
H	15.437551	-11.092727	2.595624
H	11.906954	-8.0253	4.379593
H	11.6151	-10.490871	4.477264
H	13.365634	-11.998974	3.592651
C	14.294622	-6.727381	3.23457
C	13.310436	-5.972943	2.306631
H	12.318765	-5.953744	2.769966

H	13.623669	-4.939172	2.134347
H	13.220947	-6.472633	1.33712
C	14.430347	-6.019218	4.605613
H	14.777761	-4.987716	4.499161
H	13.4554	-6.00086	5.103111
C	15.663716	-6.929853	2.56513
H	15.130203	-6.551459	5.256641
C	16.601996	-5.975028	2.15235
H	17.522005	-6.358156	1.72586
C	16.465199	-4.589163	2.256936
H	15.540817	-4.191459	2.656195
C	17.447893	-3.646657	1.904575
C	17.200538	-2.269563	2.060402
C	18.121023	-1.234551	1.774463
C	19.937805	5.812627	3.057055
C	19.258968	4.596971	3.05632
C	18.136634	4.367544	3.856299
C	17.661651	5.373308	4.687211
C	18.327485	6.605964	4.704573
C	19.450223	6.817865	3.898877
H	20.813567	5.993447	2.443214
H	16.792956	5.21403	5.320416
H	17.971645	7.401194	5.351871
H	19.958615	7.776605	3.92548
N	19.531595	3.422669	2.312391
C	17.636838	2.949329	3.631894
C	17.70417	2.139048	4.949656
H	17.055882	2.604793	5.698854
H	17.375579	1.105231	4.809106
H	18.722336	2.122677	5.349409
C	16.197121	2.975385	3.063335
H	16.15868	3.539565	2.125672
H	15.812698	1.968079	2.878507
H	15.530958	3.465594	3.780654
C	18.648581	2.430157	2.595688
C	18.753119	1.166987	2.009956
H	19.591516	1.020035	1.339089
C	17.906367	0.071062	2.232798
H	17.027773	0.228691	2.846567
C	20.645882	3.291527	1.380862
H	21.156006	4.248666	1.291459
H	21.36186	2.54436	1.739543
H	20.282046	2.997261	0.391632
C	17.096272	-8.829598	1.831571

H	17.976289	-8.564095	2.426139
H	17.005797	-9.913625	1.807011
H	17.22718	-8.46684	0.807581
C	19.386628	-1.646147	1.043841
H	20.17355	-0.903363	1.196363
H	19.20148	-1.68622	0.040314
C	18.801892	-4.084768	1.371945
H	18.705263	-4.354482	0.30901
H	19.123073	-4.996309	1.887972
C	19.882287	-3.011936	1.534404
H	20.783264	-3.306298	0.986276
H	20.161719	-2.930342	2.591926
N	15.890202	-8.257097	2.421908

Table S7 Cy structural coordinate values.

atom	X	Y	Z
C	-4.51452	2.925802	1.11796
C	-3.60505	3.870745	0.636753
C	-3.86893	5.224502	0.791193
C	-5.05201	5.614582	1.431669
C	-5.951	4.655775	1.906322
H	-6.40604	2.557885	2.130623
H	-3.17385	5.974715	0.423989
H	-5.27209	6.669738	1.560009
H	-6.86457	4.971835	2.400472
C	-2.42527	3.159365	-0.0061
C	-2.33095	3.530826	-1.50719
H	-2.17402	4.609825	-1.60431
H	-1.4992	3.023471	-2.00307
H	-3.25448	3.275014	-2.03541
C	-1.11493	3.5105	0.741796
H	-0.2492	3.000759	0.310712
H	-0.93839	4.588942	0.67765
C	-2.81725	1.685032	0.193071
H	-1.18091	3.241456	1.800378
C	-2.13535	0.526707	-0.18363
H	-2.61227	-0.41665	0.056235
C	-0.90493	0.492159	-0.85019
H	-0.44956	1.438188	-1.10672
C	-0.21104	-0.6613	-1.23786
C	0.99414	-0.5997	-1.97273
C	1.695969	-1.71814	-2.47557
C	1.660752	1.010049	-2.32021
C	7.342275	-3.93796	-6.70105

C	6.443832	-3.14741	-5.99019
C	6.545429	-1.75476	-5.94777
C	7.571211	-1.11596	-6.63033
C	8.488528	-1.89014	-7.35229
C	8.371433	-3.28227	-7.38444
H	7.265754	-5.01941	-6.73769
H	7.667222	-0.03377	-6.60944
H	9.296278	-1.40479	-7.89102
H	9.088937	-3.87035	-7.9484
N	5.324356	-3.54183	-5.21867
C	5.420629	-1.18878	-5.09586
C	6.000917	-0.43312	-3.87445
H	6.615836	0.402625	-4.22336
H	5.213808	-0.02802	-3.23297
H	6.634338	-1.08904	-3.26957
C	4.517206	-0.2644	-5.94986
H	4.101792	-0.80206	-6.80758
H	3.688598	0.14884	-5.36873
H	5.111943	0.571797	-6.33111
C	4.68604	-2.47322	-4.67441
C	3.55242	-2.62536	-3.87415
H	3.201954	-3.63899	-3.71717
C	2.836223	-1.58097	-3.27761
H	3.178972	-0.57233	-3.46033
C	4.910234	-4.92636	-5.03207
H	5.604997	-5.58398	-5.55098
H	4.912299	-5.18517	-3.96834
H	3.906223	-5.08462	-5.43922
C	-4.69537	0.396782	1.197549
H	-4.06547	-0.20943	1.856564
H	-5.61901	0.634641	1.721697
H	-4.93971	-0.18184	0.300717
C	1.146775	-3.09865	-2.14898
H	1.97585	-3.80781	-2.05386
H	0.526483	-3.45563	-2.98496
C	-0.7647	-2.03929	-0.90798
H	-1.52421	-2.31922	-1.65352
H	-1.27806	-2.00419	0.058753
C	0.328083	-3.10619	-0.85883
H	-0.11682	-4.09436	-0.70056
H	0.990291	-2.91018	-0.00619
N	-4.01224	1.632382	0.837144

Table S8 Complex excitation energy information

	State	Excitation energy/eV	oscillator strength	Wavelength/nm	
**CHO-Ir-cy**	S_1	**1.97**	**2.20750**	**628.72**	**H -> L 100%**
**Ir-cy**	S_1	**1.98**	**2.19430**	**624.89**	**H -> L 100%**
**cy**	S_1	**1.97**	**2.43610**	**630.51**	**H -> L 100%**

S1. Density functional theory calculations

All geometric optimization calculations were performed using the B3LYP functional with the 6-31G* basis set for H, C, N, and O atoms, along with the Hay-Wadt effective core potential (ECP) and double basis set LANL2DZ for iridium atoms. The calculations were conducted under vacuum conditions without any imaginary frequencies, and molecular orbital information, including energy levels and distributions, was obtained at the same theoretical level. Additionally, the vertical excitation state electronic properties of several materials were calculated based on the same functional and basis set under the implicit solvent model of DMSO. The aforementioned calculations were carried out using the Gaussian 16 C01 package. And the properties of the ground and excited state electronic wave functions were further analyzed using the Multiwfn 3.8 dev package to obtain isosurface information and to generate charge density difference plots. The spin-orbital coupling (SOC) matrix elements were evaluated with zero-order regular approximation (ZORA) Hamiltonian in scalar approximation in the ADF code.

S2. $^1O_2$ generation test with DPBF

In this study, 1,3-diphenylisobenzofuran (DPBF) was utilized as a singlet oxygen ($^1O_2$) indicator. DPBF undergoes an irreversible reaction with $^1O_2$ to form an endoperoxide, resulting in a decrease in absorbance at 415 nm. By monitoring the time-dependent reduction of DPBF absorbance at 415 nm, the singlet oxygen generation efficiency of the photosensitizer was quantitatively evaluated. Sample Preparation: a 3 mL solution containing DPBF (1 mg·mL^{-1}, 70 μL) and photosensitizer (12 μM) was prepared in a quartz cuvette; The sample was irradiated with a near-infrared LED light source (specific wavelength to be defined), absorbance at 415 nm was recorded at 10-second intervals over a 90-second period using a UV-Vis spectrophotometer.

S3. Cell culture

4T1 cells were maintained in RPMI 1640 growth medium supplemented with 10% serum and 1% penicillin and streptomycin. Cells were cultured at 37 °C in a humidified atmosphere of 95% air and 5% CO_2.

S4. Cytotoxicity test method

The cytotoxicity of **Ir-Cy** and **CHO-Ir-Cy** was assessed using the MTT assay. Briefly, 4T1 cells (1×10^4 cells per well) in 100 μL of cell suspension were seeded into a 96-well plate and incubated overnight in a humidified incubator at 37°C with 5% CO_2. After cell adherence, the original medium was removed, and 100 μL of fresh medium containing varying concentrations of **Ir-Cy** or **CHO-Ir-Cy** (0–100 μM) was added to each well. Following 6 h of incubation, the photosensitizer-containing medium was replaced with fresh blank medium. The light-treated group was irradiated with an 808 nm laser (0.50 W·cm^{-2}) for 3 min per well, while the dark group was kept under identical conditions without irradiation. After irradiation, cells were

further cultured for 24 h in the incubator. Subsequently, 10 μL of MTT solution (5 mg·mL^{-1}) was added to each well and incubated for 4 h. The medium was then replaced with 200 μL of dimethyl sulfoxide (DMSO) to dissolve the formazan crystals. The absorbance of each well at 490 nm was measured using a microplate reader.

S5. Live & dead staining test method

The live/dead cell staining assay was performed using the Calcein-AM/PI Cell Viability and Cytotoxicity Detection Kit. Briefly, 1 mL of 4T1 cell suspension (5×10^4 cells/mL) was added to confocal dishes and incubated in the dark for 24 h in a culture incubator. After incubation, photosensitizer solutions (100 μM, prepared in RPMI 1640 medium) were added to each dish, and the original medium was replaced with fresh RPMI 1640 medium following 6 h of treatment. A control group with 0 μM photosensitizer concentration was included. Subsequently, the cells were irradiated with an 808 nm laser (0.50 W·cm^{-2}) for 3 min. After irradiation, the cells were cultured overnight, followed by removal of the supernatant and two washes with phosphate-buffered saline (PBS). The cells were then stained with an appropriate volume of Calcein-AM/PI working solution (diluted in PBS) under dark conditions for 30 min. Finally, cell viability was observed using confocal laser scanning microscopy (CLSM). *S6. Evaluation of intracellular ROS production capacity*

The intracellular singlet oxygen generation capability of the photosensitizer was detected using DCFH-DA as an indicator. Briefly, 4T1 cells at a density of 5×10^4 cells/mL were seeded into confocal dishes and incubated overnight. The original medium was then replaced with fresh medium containing the photosensitizer (100 μM), followed by 6 h of incubation in a culture incubator. The light-treated group was irradiated with an 808 nm laser (0.50 W·cm^{-2})

for 2 min, while the control group (0 μM photosensitizer) was kept under the same conditions without irradiation. After treatment, the medium was removed, and the cells were incubated with RPMI 1640 medium containing DCFH-DA (10 μM) for 20 min in the dark. Subsequently, the cells were washed twice with phosphate-buffered saline (PBS), and 1 mL of PBS was added to each dish. The intracellular green fluorescence intensity, indicative of singlet oxygen production, was immediately observed using confocal laser scanning microscopy (CLSM).

S7. Test method for cell uptake capacity

The cellular uptake behavior was evaluated using confocal laser scanning microscopy (CLSM) to observe the internalization of the photosensitizer by cells. Briefly, 4T1 cells at a density of 5×10^4 cells per dish were seeded into confocal dishes and incubated overnight in a culture incubator to allow adherence. After removing the original medium, fresh medium containing the photosensitizer was added, and the cells were further incubated for 0.5 h, 2 h, or 6 h in the incubator. Following incubation, the medium was aspirated, and the cells were gently washed twice with phosphate-buffered saline (PBS). Subsequently, 1 mL of PBS was added to each dish, and the intracellular uptake of the photosensitizer was visualized under CLSM upon excitation with an 808 nm laser.
